# Central Retinal Venous Pressure in Eyes of Normal-Tension Glaucoma Patients with Optic Disc Hemorrhage

**DOI:** 10.1371/journal.pone.0127920

**Published:** 2015-05-21

**Authors:** Ko Eun Kim, Dong Myung Kim, Josef Flammer, Kyoung Nam Kim

**Affiliations:** 1 Department of Ophthalmology, Seoul National University Hospital, Seoul, Korea; 2 Department of Ophthalmology, University of Basel, Basel, Switzerland; 3 Department of Ophthalmology, Chungnam National University Hospital, Daejeon, Korea; Massachusetts Eye & Ear Infirmary, Harvard Medical School, UNITED STATES

## Abstract

**Objective:**

To compare central retinal venous pressure (CRVP) among eyes with and without optic disc hemorrhage (ODH) in bilateral normal-tension glaucoma (NTG) patients and NTG eyes without an episode of ODH.

**Methods:**

In this prospective study, 22 bilateral NTG patients showing a unilateral ODH and 29 bilateral NTG patients without an episode of ODH were included. Eyes were categorized into group A (n = 22, eyes with ODH), group B (n = 22, fellow eyes without ODH), and group C (n = 29, NTG eyes without an episode of ODH). A contact lens ophthalmodynamometer was used to measure CRVP and central retinal arterial pressure (CRAP).

**Results:**

Intraocular pressure (IOP) measured on the day of contact lens ophthalmodynamometry showed no difference among groups. However, the mean baseline IOP in group A was significantly lower than that in group C (*P *= .008). The CRVP in group A (29.1 ± 10.8 mmHg) was significantly lower than that in group C (40.1 ± 8.8 mmHg, *P* = .001), but similar to that in group B (30.5 ± 8.7 mmHg, *P* = .409). A similar relationship was noted for CRAP. No significant eye-associated variable for ODH was found in group A and B by conditional logistic regression analysis (all *P* > 0.05). However, multivariate logistic regression analysis in groups A and C revealed that low mean baseline IOP (odds ratio [OR] = 0.69, 95% confidence interval [CI] 0.49-0.98, *P* = 0.043) and low CRVP (OR = 0.88, 95% CI 0.80-0.95, *P* = 0.003) were associated with ODH.

**Conclusions:**

CRVP was lower in NTG eyes with ODH than in eyes without an episode of ODH, but similar to that of fellow eyes without ODH. These imply less likelihood of association between increased central retinal venous resistance and ODH.

## Introduction

Optic disc hemorrhage (ODH) has been demonstrated to have a strong association with the development [1–3] and progression [4–8] of glaucoma. Previous studies have revealed that ODH may occur due to mechanical microvascular disruption incurred from alterations of the lamina cribrosa,[9] ischemic microinfarction at the optic disc,[10,11] or high translaminar pressure gradient-related stress on the optic nerve head.[12,13] Although there has been large number of studies to reveal the pathomechanism of ODH, much remains to be elucidated at present.

In glaucomatous eyes, retinal veins in the immediate vicinity of, as well as away from the optic disc, demonstrated lower pulse amplitudes than in healthy eyes, indicating disturbances in venous outflow and increased intraluminal venous pressure and resistance.[14,15] Morgan et al. suggested that such a gradual increase in central retinal vein resistance may be implicated in central retinal vein occlusions, shunt vessels, and ODHs.[12] In addition, ODH occurs up to 4 times more frequently in normal-tension glaucoma (NTG) than in primary open-angle glaucoma (POAG) patients with baseline intraocular pressure (IOP) of more than 21 mmHg.[7,16,17] Increase in the pressure and resistance of the peripheral vessels, resulting in inadequate perfusion of the proximal portion of the optic nerve, has been suggested as the main theory for explaining such a difference.[18] However, there has been no investigation directly assessing the arterial and venous blood pressures of the optic nerve head in glaucoma patients with ODH.

This study aimed to compare the central retinal venous pressure (CRVP) and central retinal arterial pressure (CRAP) among eyes with and without ODH in the same patients with bilateral NTG and eyes of NTG patients without an episode of ODH by using a contact lens ophthalmodynamometer. To the best of our knowledge, this is the first study to measure the CRVP and CRAP in glaucomatous eyes with ODH in the standard pressure unit, mmHg.

## Patients and Methods

This is a prospective, comparative clinical study including bilateral NTG patients who visited the Glaucoma Clinic of Seoul National University Hospital between August 2012 and May 2014. The study enrolled consecutive (1) bilateral NTG patients showing unilateral ODH and (2) bilateral NTG patients without an episode of ODH in any eye during a follow-up period of more than 5 years before enrollment. Each subject was informed about the nature of the study, and written informed consent was received from all the participants after study approval by the institutional review board of Seoul National University Hospital Biomedical Research Institute. This study conduct adhered to the Declaration of Helsinki.

### Subjects

All participants had completed ophthalmic examinations, including measurement of best-corrected visual acuity and spherical equivalent of refraction, IOP measurement by Goldmann applanation tonometer, slit-lamp biomicroscopy, gonioscopy, dilated fundus examination, and central corneal thickness measurement using pachymetry (Pocket II Pachymeter Echo graph; Quantel Medical, Clermont-Ferrand, France). Diurnal IOP was defined as IOP measurements obtained at 8:30 AM, 10:00 AM, 11:30 AM, 1:00 PM, 2:30 PM, and 4:00 PM before initiating first medication and their averaged value was defined as mean baseline IOP. The patients underwent stereo optic disc photography, red-free fundus photography (Vx-10; Kowa Optimed, Tokyo, Japan), and standard automated perimetry using Swedish interactive threshold algorithm 30–2 standard program (Humphrey Field Analyzer II; Carl Zeiss Meditec Inc., Dublin, CA).

Inclusion criteria were best-corrected visual acuity of 20/40 or better, spherical equivalent within ± 5 diopters, and cylindrical correction within ± 3 diopters. For enrollment, participants should have been diagnosed as having bilateral NTG and should have shown well-control of IOP with topical IOP-lowering medications. For NTG diagnosis, patients should have an open anterior chamber angle, a glaucomatous optic disc appearance (e.g., neuroretinal rim thinning, excavation, retinal nerve fiber layer defect), a glaucomatous visual field defect, and a baseline diurnal IOP of same or less than 21 mmHg. A glaucomatous visual field defect was defined as follows: (1) the presence of a cluster of 3 or more non-edge points with a probability of occurrence of < 5% in the normal population on the pattern deviation plot, with one of these points having the probability of occurrence of < 1% of the normal population, (2) a pattern standard deviation of < 5%, or (3) glaucoma hemifield test results outside normal limits. Only participants with reliable visual fields (fixation loss < 20%, false-positive and negative errors < 15%) and glaucomatous visual field defect confirmed by repeated visual field tests within a month were included. Eyes of bilateral NTG patients were categorized into 3 groups according to the presence or absence of unilateral ODH at enrollment: (1) group A (n = 22; NTG eyes showing ODH at the time when contact lens ophthalmodynamometry [CLOD] was performed); (2) group B (n = 22; fellow eyes of group A not showing ODH); and (3) group C (n = 29; eyes of bilateral NTG patients without an episode of ODH serving as control). One eye was randomly selected for the inclusion in group C. Systemic medical treatment was continued. All patients were followed-up every 3 or 4 months at the Glaucoma Clinic of Seoul National University Hospital as long as there was no ODH. If an ODH was detected by fundoscopy or stereo optic disc photography at the regular follow-up, the patients were followed-up every 3 to 4 weeks until it disappeared. After the disappearance, patients were seen 1 month later for confirmation and then every 3 or 4 months. In group C, a patient with a mean deviation of Humphrey central 30–2 threshold visual field greater than -12 dB was excluded because ODH occurred more frequently in the early to moderate stage of glaucoma, and rarely in its advanced stage.[19]

For all participants included in the study, blood pressure (BP) was measured using a digital automatic BP monitor (Omron HEM-770A; Omron Matsusaka Co., Ltd., Matsusaka, Japan) after they had seated and rested for 5 minutes. BP was measured twice at intervals of 5 minutes apart and a third measurement was made if systolic BP (SBP) differed by more than 10 mmHg or diastolic BP (DBP) differed by more than 5 mmHg.[20] Mean arterial pressure (MAP) was defined as DBP + 1/3(SBP—DBP). Ocular perfusion pressure (OPP) was defined as 2/3MAP—IOP.[21]

Exclusion criteria were participants that had caffeine intake, smoking, or exercise at the day of CLOD, those showing non-glaucomatous optic neuropathy (e.g., hereditary optic neuropathy, ischemic optic neuropathy, optic neuritis), any disease that can affect the visual field (e.g., retinal vascular occlusive disease, diabetic retinopathy, brain tumor), and history of any ocular surgery other than simple cataract surgery.

### Contact lens ophthalmodynamometer examination

The FDA-approved Contact Lens Ophthalmodynamometer (Meditron GmbH, Voelklingen, Germany) was used to measure the CRVP and CRAP.[22–26] The instrument consists of a standard 3-mirror Goldmann contact lens with a measuring device on its rear side, which contains several precision sensors. The sensors continuously measure the force that the ophthalmologist exerts on the eye by means of the contact lens. The instrument is connected by a thin flexible cable with a portable central unit. The increase in IOP calculated from the compressive pressure of the contact lens is shown in mmHg on the liquid crystal display screen of the central unit.

Participants were in a sitting position, and the chin and forehead were placed on the slit-lamp biomicroscope set at 16-fold magnification. Before performing CLOD, the optic disc was first examined using slit-lamp biomicroscope and noncontact 90 D indirect ophthalmic lens. The central retinal vessels and their branches on the optic disc surface were carefully examined for the presence of spontaneous venous pulsation (SVP). After instillation of one drop of 0.5% proparacaine solution, (Paracaine; Hanmi Pharm Co., Ltd., Seoul, Korea), a topical anesthesia, the Goldmann contact lens of the Contact Lens Ophthalmodynamometer was gently placed onto the eye with as little pressure as possible with 2% hydroxypropylmethylcellulose gel (Hycell solution; Samil Pharm Co., Ltd., Seoul, Korea) as a contact fluid. After placement of the contact lens on the eye, the optic disc was continuously monitored while gradually increasing the compressive force. When the central retinal vein or one of its branches on the optic disc surface started to show pulsation, the value provided by the pressure sensor was fixed and considered as the diastolic CRVP. The compressive force was then released, and this measurement cycle was repeated another 3 times. The first measurement was performed to feel and check the approximate compressive forces in each patient, and thus, it was not included in the analysis. The following 3 values of pressure were averaged and used for analysis. After repeated measurements of CRVP, the compressive pressure increased until the central retinal artery or one of its branches on the optic disc surface showed pulsation. The value measured at that moment was considered to be the diastolic CRAP. The arterial pressure measurements were performed in the same manner as the venous pressure. Finally, the IOP was added to the ophthalmodynamometric measurements since both the IOP and the pressure asserted onto the globe through the contact lens acted as a sum to provoke pulsation of a vessel. If a SVP was noticed, CRVP was considered to be equal to IOP, and CRAP measurement was not performed.

### Statistical analysis

The baseline characteristics among groups were compared using Wilcoxon signed rank test (group A vs. B) or Mann-Whitney U test (group C vs. A or B) for continuous variables, and chi-square test or Fisher’s exact test for categorical variables. Pressure measurements between groups were compared using Wilcoxon signed rank test (group A vs. B) or Mann-Whitney U test (group C vs. A or B). While comparing CRAP, we excluded 2 eyes from group B whose CRAP measurements were not performed due to SVP. In our study which unilateral ODH patients were intentionally included, a strongly negative correlation in the occurrence of ODH exists between eyes with ODH and the fellow eyes without ODH. In such binary data with negative correlation and possibility of selection bias, the method of generalized estimating equations (GEE),[27] a commonly used approach to analyze correlated data, cannot assure reliability.[28,29] Therefore, separate group analyses were performed instead to determine variables associated with ODH: conditional logistic regression analysis was conducted in group A and B to find an eye-associated variable and logistic regression analysis in group A and C for an individual variable. After univariate logistic regression analysis, variables with *P* < 0.10 were entered into multivariate logistic regression analysis by using stepwise selection method. Odds ratio (OR) with 95% confidence interval (CI) was used to represent the association. Statistical analyses were performed using SPSS version 21.0 (IBM Corp., Armonk, NY) and SAS version 9.2 (SAS Inc., Cary, NC). Statistical significance was defined as *P* < 0.05. The required sample size for each group was 18 using PASS version 18.0 (NCSS LLC., East Kaysville, UT) for the following conditions: two-sided test; common standard deviation, 10.8 mmHg; clinically relevant difference, 11.0 mmHg; α = 0.05; and power, 90%.

## Results

Twenty-eight patients showing unilateral ODH and 31 patients with no episode of ODH during the recent follow-up period were initially enrolled. Two patients were excluded because of unreliable examination and 6 patients refused to undergo CLOD. Finally, 22 NTG patients with unilateral ODH (group A and B) and 29 NTG patients without an ODH episode in any eye during the recent 5 years of follow-up (group C) were included in the analyses.

The baseline characteristics of included patients are summarized in Table 1. Twelve (54.5%) of 22 eyes in group A had recurrent ODH in the same eye in a recent year from the time of enrollment. No significant differences were found between the 2 groups in age, gender ratio, SBP, DBP, pulse rate, number of patients with systemic co-morbidities, and distribution of IOP-lowering topical medications.

**Table 1:** Comparison of baseline characteristics between eyes of bilateral normal-tension glaucoma patients with unilateral optic disc hemorrhage (ODH group) and those of bilateral normal-tension glaucoma patients without an episode of optic disc hemorrhage (Non-ODH group).

	ODH group (n = 22)	Non-ODH group (n = 29)	*P*
**Age (years)**	54.6 ± 12.8	53.2 ± 12.5	0.703*
**Male (n, %)**	6 (27.3)	8 (27.6)	0.980^†^
**Right eye (n, %)**	11 (50.0)	15 (51.7)	0.903^†^
**Systemic blood pressure (mmHg)**			
Systolic	119.8 ± 10.3	116.1 ± 11.0	0.106*
Diastolic	77.5 ± 9.0	73.7 ± 6.5	0.101*
Mean arterial pressure	91.6 ± 9.0	87.9 ± 6.3	0.121*
**Pulse rate (beats/minute)**	66.3 ± 7.5	67.0 ± 7.7	0.710*
**Systemic conditions (n, %)**			
Self-declared cold hands/feet	6 (27.3)	9 (31.0)	0.770^†^
Hyperthyroidism	1 (4.5)	1 (3.4)	1.000^‡^
Hypertension	6 (27.2)	5 (17.2)	0.498^‡^
Hypercholesterolemia	1 (4.5)	0	0.431^‡^
Cardiac disease	2 (9.1)	1 (3.4)	0.571^‡^
Diabetes mellitus	1 (4.5)	0	0.431^‡^
Sleep apnea syndrome	0	1 (3.4)	1.000^‡^
**IOP-lowering topical medications** ^**§**^			0.389^‡^
Alpha agonists	3	6	
Beta-blockers	10	17	
Prostaglandin analogs	16	18	
Carbonic anhydrase inhibitors	12	8	

IOP = intraocular pressure

*Mann-Whitney U test

^†^Chi-square test

^‡^Fisher’s exact test

^§^Patients treated with more than one drug or fixed combination drugs were multiply counted according to the number of used drugs.

Data are represented as mean ± standard deviation unless otherwise noted.

**Fig 1:**
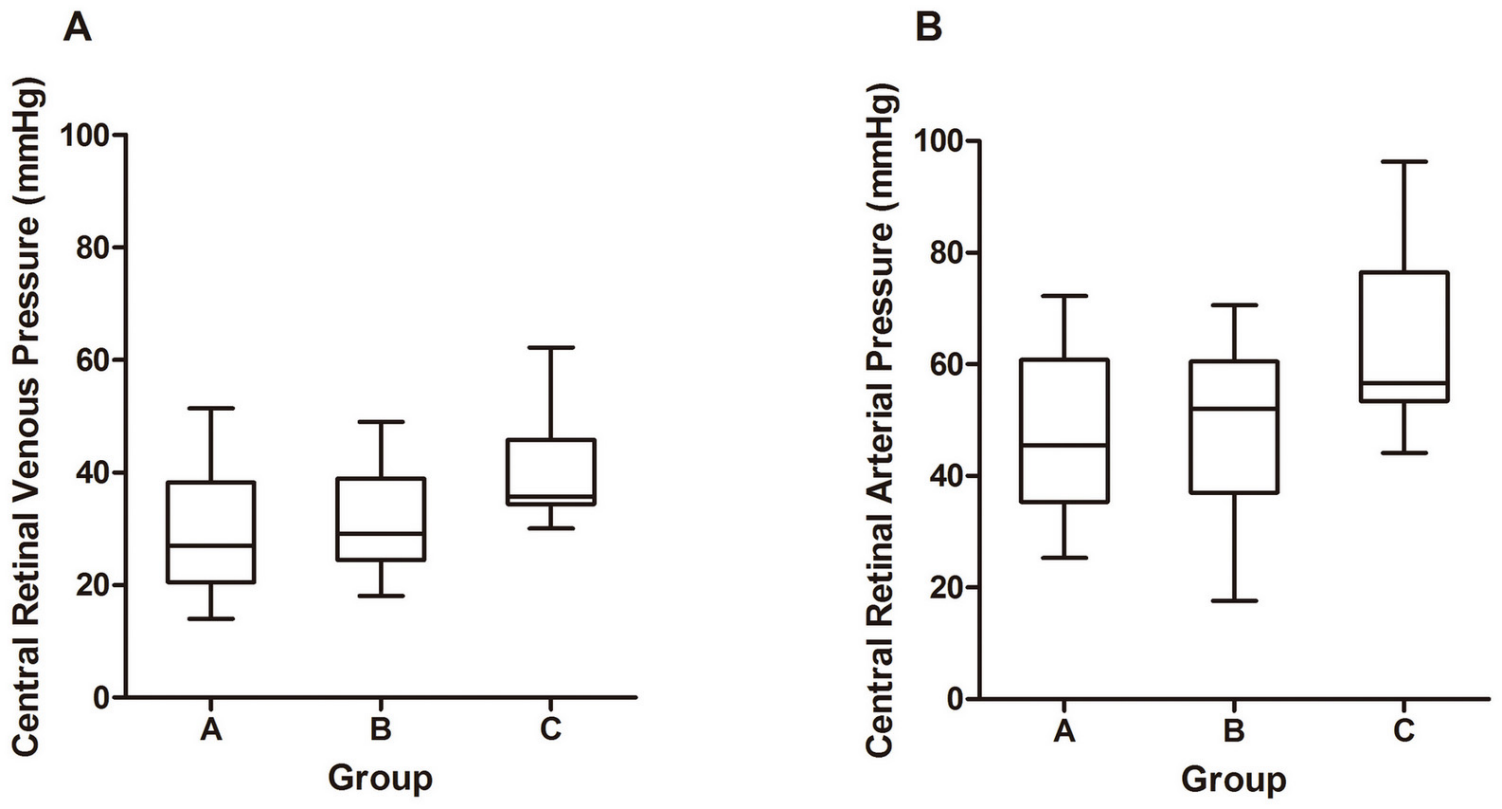
The distribution of the pressure in the (A) central retinal vein and (B) central retinal artery measured by using contact lens ophthalmodynamometer in normal-tension glaucoma (NTG) patients with and without optic disc hemorrhage (ODH): (1) group A (eyes of bilateral NTG patients with ODH); (2) group B (fellow eyes with no ODH); (3) group C (randomly selected eyes of bilateral NTG patients without an episode of ODH).

Table 2 presents the comparison of ocular parameters and pressure measurements among the 3 groups. There was no statistically significant difference among the groups in spherical equivalent of refraction, central corneal thickness, or visual field indices. The IOP measured on the day of CLOD was 11.4 ± 1.5 mmHg in group A, 11.6 ± 2.4 mmHg in group B, and 11.8 ± 1.8 mmHg in group C, which showed no difference. However, the mean baseline IOP in group A (13.3 ± 1.3 mmHg) was significantly lower than that in group C (14.7 ± 2.4 mmHg; *P* = 0.008), and so were the lowest (11.8 ± 1.3 vs.13.2 ± 2.2 mmHg; *P* = 0.009) and the highest pressures of baseline diurnal IOP (14.7 ± 1.6 vs.16.2 ± 2.7 mmHg; *P* = 0.011). Interestingly, the CRVP of group A was 29.1 ± 10.8 mmHg, which was significantly lower than that in group C (40.1 ± 8.8 mmHg; *P* = 0.001), but no difference was found in comparison with fellow eyes in group B (30.5 ± 8.7 mmHg; *P* = 0.409; Fig 1A). CRAP values in group A and B were also lower than that in group C (Fig 1B). The difference between MAP and CRAP, which implies flow resistivity, was significantly greater in group A (42.4 ± 13.5 mmHg) than in group C (26.6 ± 14.4 mmHg; *P* = 0.001), but similar to that of fellow eyes in group B (41.9 ± 17.0 mmHg; *P* = 0.841). For OPP, no difference was found between groups.

**Table 2:** Comparison of characteristics among eyes of normal-tension glaucoma patients with unilateral optic disc hemorrhage (group A), fellow eyes without optic disc hemorrhage (group B), and eyes of normal-tension glaucoma patients without an episode of optic disc hemorrhage (group C)

	Group A (n = 22)	Group B (n = 22)	Group C (n = 29)	*P*_*A-B*_ *	*P*_*A-C*_ ^†^	*P*_*B-C*_ ^†^
**Spherical equivalent of refraction (diopters)**	-2.20 ± 3.22	-2.12 ± 2.99	-2.90 ± 3.31	0.955	0.457	0.455
**Central corneal thickness (μm)**	529.9 ± 29.2	531.5 ± 30.6	530.1 ± 36.4	0.226	0.920	0.631
**Diurnal intraocular pressure (mmHg)**						
Lowest	11.8 ± 1.3	12.3 ± 1.7	13.2 ± 2.2	0.296	**0.009**	0.119
Highest	14.7 ± 1.6	15.2 ± 2.2	16.2 ± 2.7	0.071	**0.011**	0.128
**Mean baseline intraocular pressure (mmHg)**	13.3 ± 1.3	13.7 ± 1.9	14.7 ± 2.4	0.086	**0.008**	0.113
**Intraocular pressure (mmHg)** ^**‡**^	11.4 ± 1.5	11.6 ± 2.4	11.8 ± 1.8	0.614	0.339	0.536
**Humphrey C30-2 threshold visual field**						
Mean deviation (dB)	-4.08 ± 3.58	-4.70 ± 3.94	-3.16 ± 2.35	0.687	0.621	0.393
Pattern standard deviation (dB)	6.88 ± 5.09	7.38 ± 5.24	5.71 ± 3.25	0.658	0.697	0.411
**Central retinal venous pressure (mmHg)**	29.1 ± 10.8	30.5 ± 8.7	40.1 ± 8.8	0.409	**0.001**	**<0.001**
**Central retinal arterial pressure (mmHg)** ^**§**^	48.0 ± 13.9	49.1 ± 15.9	63.2 ± 14.6	0.841	**0.003**	**0.016**
**Ocular perfusion pressure (mmHg)**	49.7 ± 6.4	48.9 ± 6.7	45.8 ± 5.5	0.411	0.130	0.217

*Comparison performed using Wilcoxon signed rank test

^†^Comparison performed using Mann-Whitney U test

^‡^Intraocular pressures measured on the day of contact lens ophthalmodynamometry

^§^As central retinal arterial pressure measurements were not conducted in 2 eyes from group B due to presence of spontaneous venous pulsation, only 20 eyes were included in the comparison between eyes in group B versus A or C.

Data are represented as mean ± standard deviation unless otherwise noted.

Significant values with *P* < 0.05 are in bold.

No significant eye-associated variable for ODH was found in group A and B by conditional univariate logistic regression analysis. However, univariate logistic regression analysis in group A and C determined that following variables were significantly associated with ODH (Table 3): mean baseline IOP (OR = 0.67, 95% CI 0.48–0.94, *P* = 0.020), CRVP (OR = 0.88, 95% CI 0.81–0.96, *P* = 0.002), and CRAP (OR = 0.93, 95% CI 0.89–0.98, *P* = 0.007). After univariate analysis, variables with *P* < 0.10, including mean baseline IOP, CRVP, CRAP, MAP, and OPP remained in the multivariate analysis. Consequently, low mean baseline IOP (OR = 0.69, 95% CI 0.49–0.98, *P* = 0.043) and low CRVP (OR = 0.88, 95% CI = 0.80–0.95, *P* = 0.003) were associated with ODH by stepwise selection method.

**Table 3:** Variables significantly associated with optic disc hemorrhage in eyes of normal-tension glaucoma patients with unilateral optic disc hemorrhage (group A) and those without an episode of optic disc hemorrhage (group C) by univariate and multivariate logistic regression analyses.

		Odds ratio (95% CI)	*P* *
Univariate analysis	Mean baseline intraocular pressure	0.67 (0.48–0.94)	0.020
	Central retinal venous pressure	0.88 (0.81–0.96)	0.002
	Central retinal arterial pressure	0.93 (0.89–0.98)	0.007
Multivariate analysis	Mean baseline intraocular pressure	0.69 (0.49–0.98)	0.043
	Central retinal venous pressure	0.88 (0.80–0.95)	0.003

CI = confidence interval

*Statistical significance was defined as *P* < 0.05.

## Discussion

The strong association between ODH and glaucoma has been demonstrated by a number of studies.[1–8] However, the pathomechanism of ODH development, the mechanism by which ODH affects the structural progression of glaucoma, or the predisposing conditions for ODH are still debatable. Among the pathomechanisms for ODH in glaucoma, elevated resistance and pressure in the central retinal vein has been proposed in consideration of ischemic theory. In this regard, we sought to investigate whether the CRVP is actually higher in NTG patients with ODH using CLOD, measured in mmHg. The present study showed similar CRVP in eyes of the same NTG patients with unilateral ODH regardless of presence of ODH. However, the CRVP was lower in eyes showing ODH compared to those of NTG patients without an ODH episode.

It is suspected that in glaucoma, with time, a venous wall thickening in response to higher shear occurs.[12] Furthermore, in the experimental glaucoma model, the area encompassing the central vascular trunk showed markedly higher connective tissue volume fraction in distinct regions.[30] These may be possible explanations to account for the increased venous resistance in glaucoma, and its association with ODH. However, others have reported findings that are contrary to such an association. In a recent study by Pillunat et al.,[26] moderate and advanced glaucoma groups presented higher CRVP compared to the early glaucoma group and controls. However, ODH is more frequently found in earlier stages of glaucoma,[19,31] which does not show exact inverse relationship with CRVP. Secondly, the frequency of SVP is reported to be lower in glaucoma patients compared to healthy individuals due to increased resistance of the central retinal venous drainage.[32,33] In this regard, absence of SVP is expected to be more frequent in glaucoma patients with ODH compared to those without ODH. However, Kim et al.[34] found that there was no significant difference in the frequency of SVP between glaucoma eyes with and without ODH. Moreover, they found no significant association between the SVP and ODH.[34] These results indicate that ODH development may not be associated with increased venous resistance and the present study shares common aspects with them.

Previously, Balaratnasingam et al.[35] reported no significant association between ODH and ophthalmodynamometric force, but the number of patients with ODH (n = 9) was small. In addition, ophthalmodynamometric measurements were assessed in an arbitrary unit, thus comparing them with conventional pressure measurements were impossible. In our independent population with larger number of NTG patients with and without ODH, central retinal vascular pressures in NTG eyes with ODH were significantly lower than those in NTG eyes without a hemorrhagic episode. Then, a question is raised on why NTG eyes with ODH showed lower CRVP than those without ODH. First, NTG eyes with ODH are assumed to have higher resistivity to blood flow based on their greater difference between MAP and CRAP. Such higher flow resistivity may lead to low ocular inflow and outflow. Primary vascular dysregulation could be one of the possible causes, suggestive of underlying ischemic condition in NTG eyes with ODH.[36] Second, in regard to recent evidences showing the role of cerebrospinal fluid pressure (CSFP) in the pathophysiology of NTG,[37] we postulated that NTG eyes with ODH may have low CSFP and in turn, low CRVP. Recently, Chen et al.[38] reported a case of white woman with NTG who had CSFP reduction by a ventriculoperitoneal shunt and had postoperative progressive visual field loss and newly onset ODH. Similar results were also found in Yang et al.[39]’s non-human primate experimental glaucoma models. Furthermore, Querfurth et al.[40] reported a linear relationship between the venous outflow pressure of the central retinal vein and CSFP. Therefore, data from these studies support our hypothesis that if NTG eyes with ODH have lower CSFP than those without ODH, their CRVP may show the same relationship. Nevertheless, ODH is a complex-cause phenomenon and our explanations still have limitations in further explaining the similar CRVP level between ODH-eye and non-ODH-eye in the same NTG patient.

The CRVP and CRAP measurements of ODH-eyes were compared with not only non-ODH-eyes, but also with fellow eyes without ODH. We believe that this study design enabled our investigation under the control of systemic confounding factors. Given that the central retinal vascular pressures in eyes with ODH were similar to those in fellow eyes without ODH, we speculate that there may be an individual predilection for ODH. This may partly explain why ODH does not develop in all glaucoma patients and why some patients have recurrent ODH.[16] There was no difference in co-morbidities between groups, but this could be due to limited data based on self-report information. Further investigations with more detailed systemic evaluations and a systematic questionnaire may be warranted to identify individual vascular risk factors for ODH.

In the present study, ODH group had slightly higher SBP and DBP measurements compared to non-ODH group, but the difference was insignificant. The association between ODH and systemic hypertension is still controversial: there have been studies that have found a relationship between systemic hypertension and ODH,[17,41,42] while others have not.[43,44] Although the BP measurements in this study had no significant association with ODH, further studies with large number of patients having a wide range of BP measurements are required to fully address this question.

Our study has several limitations. First, based on the fact that ODH usually persists from 2 to 35 weeks, with an average of 11 weeks,[16] we might not have detected some cases of ODH in group B and C during 3–4 months of regular follow up. Nevertheless, a clear difference in CRVP was observed between fellow eyes without ODH and the control NTG eyes. One may also argue that the central retinal vascular pressures measured in this study may reflect the post-hemorrhage pressures rather than the pressures at the moment of bleeding. We cannot exclude that the central retinal vascular pressures in group A were higher than those in group C at the moment of or just before the bleeding. However, practically, we cannot detect the time of bleeding. In addition, if increase in vascular resistance were to play a role in the development of ODH, then it would remain constant even after hemorrhagic event and thus, pressures in eyes with ODH would be at least higher than those without ODH. Second, one confounding factor that needs to be taken into account is that all medications were continued. Certain classes of medications can potentially interfere with the ocular venous tone. However, none of the patients were taking calcium channel blockers, which may block the vasoactive response of endothelin,[45] or anti-platelet drugs, which could increase the risk of developing ODH.[43] Third, since the moment of the first vessel wall movement was taken as a measurement point for an early collapse, it is not clear whether vessel collapse pressure differs between the hemisphere where ODH is present and the contralateral hemisphere. Lastly, only NTG patients with baseline IOP of same or less than 21 mmHg were included. Thus, the general application of our results to POAG patients with high baseline IOP may have limits. However, in our study, even NTG patients, who have been thought to show a greater association with vascular risk factors than POAG patients with high baseline IOP, showed negative results.

In conclusion, the CRVP was not increased in NTG patients with ODH, and was even lower compared to that in NTG patients without an episode of ODH. Our findings may not provide the direct evidence, but at least they do not support the association between increased venous resistance and ODH in NTG patients. In addition, based on similar pressure measurements between NTG eyes with ODH and with fellow NTG eyes without ODH, the predisposing other intrinsic vascular condition may be associated with ODH development. Further investigations in larger number of patients with different baseline IOP is required to determine such factors to understand individual predilections for ODH and the pathomechanism of ODH in glaucoma.
